# Rehabilitation of a 13‐Year History of Habitual Tic‐Induced Nail Dystrophy

**DOI:** 10.1111/jocd.16641

**Published:** 2024-11-11

**Authors:** Liang Chen, Zheyuan Li, Tianyi Liu

**Affiliations:** ^1^ Shanghai Key Laboratory of Clinical Geriatric Medicine, Huadong Hospital Shanghai China; ^2^ Department of Plastic and Aesthetic Surgery, Huadong Hospital, Shanghai Medical College Fudan University Shanghai China; ^3^ Intensive Care Unit, Zhongshan Hospital Fudan University Shanghai China

**Keywords:** case report, habitual tic‐induced nail dystrophy, nail disorders, onychotillomania

## Abstract

**Background:**

Habitual tic nail dystrophy is a prevalent condition among adults; however, it has received limited scholarly attention.

**Aims:**

This study aims to report the case of habitual tic nail dystrophy with a duration of 13 years.

**Methods:**

The patient was advised to modify his lifestyle habits and was followed up regularly over the course of 1 year to assess changes in the appearance of his nails.

**Results:**

The appearance of the nails normalized during the 1‐year follow‐up period.

**Conclusions:**

The appearance of nails in patients with habitual tic nail dystrophy can be significantly improved through lifestyle modifications and by minimizing the stimulation of the nail matrix.

## 1 Introduction

Nail dystrophy refers to an abnormal structure of nails, which may manifest as ridges, pitting, lack of luster, roughness, and fragility; these anomalies can lead to both health and aesthetic concerns. The habitual tic deformity is due to long‐term repeated friction or stimulation of the nail plate, especially near the deck and near the proximal nail fold repeated stimulation and friction, which leads to a central linear depression of the nail plate. This condition is often accompanied by parallel transverse ridges extending from the proximal to the distal end of the nail, as well as the disappearance of the cuticle and/or the presence of a prominent half‐moon appearance [1]. Collectively, the nails may resemble a washboard, hence the term “washboard nails” [2]. This condition commonly affects the thumbnails and may involve both thumbs. Dermoscopic examination may reveal transverse grooves, periungual scales, prominent half‐moons, longitudinal grooves, branching grooves, reddening, and hemorrhage in the proximal nail fold [3]. This disorder falls under the broad category of nail tic disorders [2], is prevalent among adults, and is largely considered a subconscious behavior [2]. To date, only a limited number of studies have focused on habitual tic‐induced nail dystrophy, with even fewer documenting its treatment and recovery process. Therefore, this article presents the case study detailing a 13‐year history of habitual tic‐induced nail dystrophy, including the treatment and recovery journey.

## 2 Case Report

**FIGURE 1:**
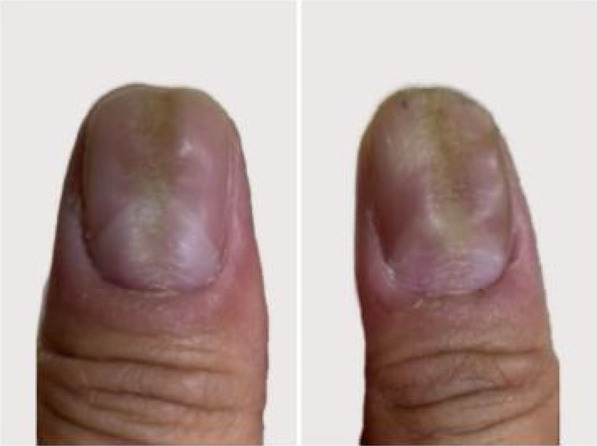
Pretreatment‐Frontal view.

**FIGURE 2:**
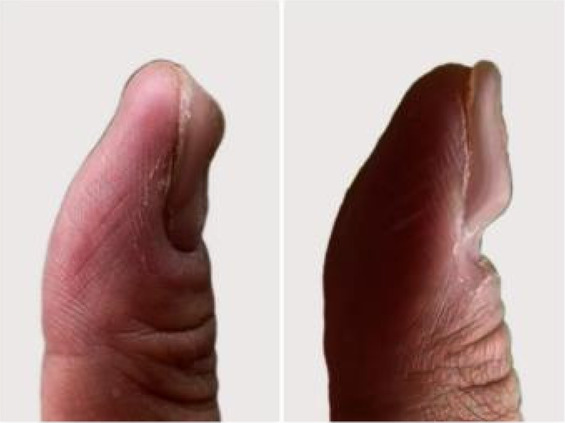
Pretreatment‐Lateral view.

**FIGURE 3:**
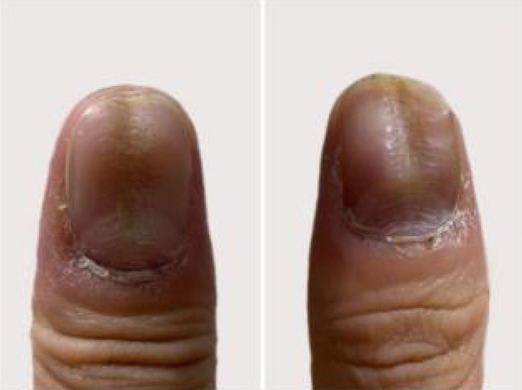
Half‐a‐month.

**FIGURE 4:**
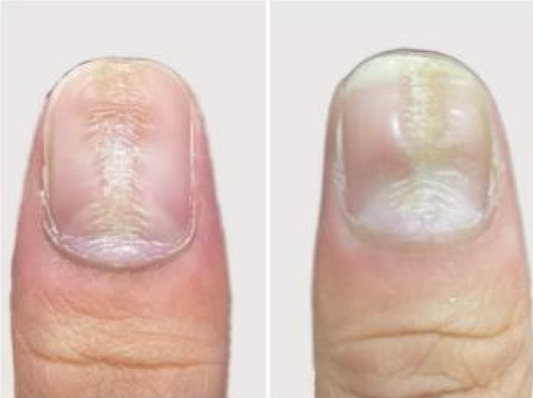
One month.

**FIGURE 5:**
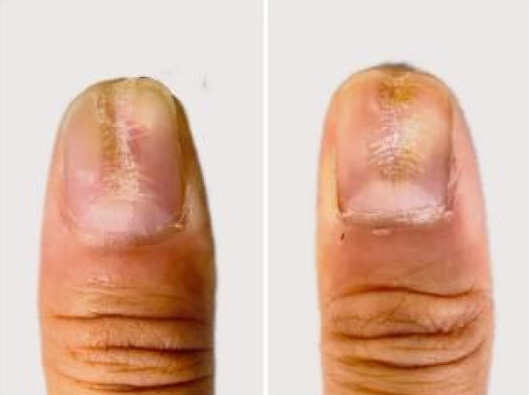
Two months.

**FIGURE 6:**
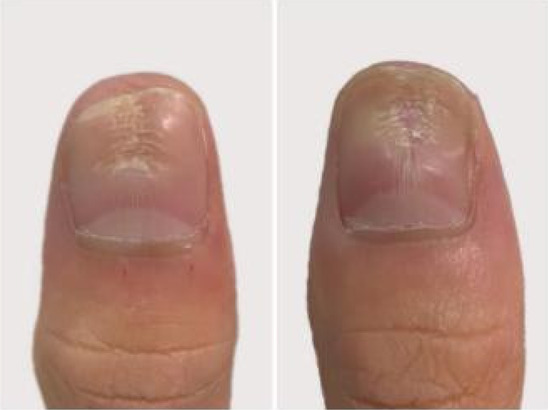
Three months.

**FIGURE 7:**
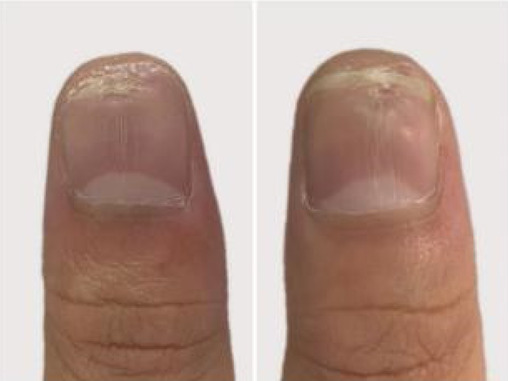
Five months.

**FIGURE 8:**
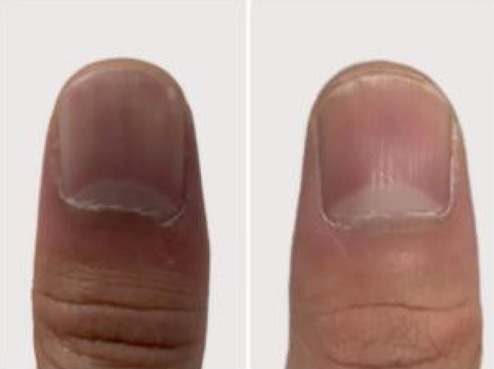
Eleven months.

A 32‐year‐old male presented for medical evaluation with a 13‐year history of abnormal nail plates on both thumbnails, which he reported as severely impacting his social activities. Upon examination, the patient's thumbnails exhibited multiple transverse ridges, accompanied by longitudinal defects and a prominent half‐moon shape, with the cuticle absent (Figure 1). Lateral views revealed an elevation at the anterior end of the left thumbnail and a mid‐posterior elevation of the right thumbnail, alongside a sunken appearance in the periungual area (Figure 2). During the interview, the patient acknowledged an involuntary habit of rubbing the base of the thumbnails with his index or middle finger, a behavior that coincided with the onset of the nail plate deformity. The patient had no history of substance abuse, systemic disease, or psychological disorders. He has previously undergone antifungal therapy, but there has been no improvement. There was no history of chemical exposure, occupational contact, or nail trauma. He was diagnosed with habitual tic‐induced nail dystrophy. Following the consultation, the patient was advised to modify his habits by consciously reducing friction at the nail bases and was instructed to return for follow‐up every half to 1 month. Over a 1‐year follow‐up period, with only a change in habits and no medical treatment, there was a significant improvement in the appearance of the nails (Figures 3, 4, 5, 6, 7, 8). This confirmed that the nail deformity was associated with chronic frictional trauma at the local nail root area.

## 3 Discussion

Habitual tic‐induced nail dystrophy may appear to be a neglected condition; however, this habitual behavior can lead to chronic traumatic injury to the nail root, resulting in making the nail matrix corresponding to the injured nail root produce sustained inflammation, and eventually manifests as nail dystrophy. Reports indicate that habitual tic‐induced nail dystrophy can lead to complications, including frictional melanonychia, infections, and permanent nail dystrophy [2, 4, 5], all of which can adversely affect the patient's health and social interactions. Furthermore, accurate diagnosis of this condition requires differentiation from median canaliform nail dystrophy, a rarer condition characterized by a split along the midline of the nail. This condition manifests as oblique ridges and a pine‐tree‐like central canaliform dystrophy of the nail surface, while the cuticle at the proximal nail fold may appear normal [4].

Habitual tic‐induced nail dystrophy is primarily characterized as a daily habit, which may be accompanied by mild anxiety in some patients. However, this condition is rarely associated with psychiatric comorbidities such as obsessive–compulsive disorder. The improvement of this symptom largely involves guiding patients to modify their behavioral habits and to avoid friction against the root of the nail plate [2]. Previous reports on the treatment of this condition include both nonpharmacological and pharmacological approaches. Nonpharmacological treatment options include the application of a gentle ointment, massaged from the proximal to the distal end of the nail, applied three times daily, which has been shown to be effective for two‐thirds of patients [6], or the use of cyanoacrylate adhesive applied to the proximal nail fold to create a sustainable barrier to trauma and to artificially reconstruct the missing cuticle, which has also proven to be a useful and cost‐effective treatment option [7]. Some scholars have suggested that a 0.2% benzalkonium chloride solution is effective in treating habitual nail deformities, as it protects the cuticle while promoting improvement in the patient's habit [5]. For patients with psychiatric comorbidities, cognitive behavioral therapy and oral medications, such as selective serotonin reuptake inhibitors (SSRIs), tricyclic antidepressants, or antipsychotics, may be considered [8]. Trials involving SSRIs or habit reversal therapy have demonstrated a positive impact on disease improvement [9, 10].

## 4 Conclusions

This article presents the case of a patient with habitual tic‐induced nail dystrophy, confirming that merely altering the patient's lifestyle habits and reducing friction on the nail plate can effectively alleviate this symptom. The detailed documentation of the patient's recovery process not only provides valuable insights for clinical healthcare professionals in diagnosis and treatment but also plays a significant role in rehabilitation guidance and fostering healing confidence among similar patients.

## Ethics Statement

All information in this article complies with the code of medical ethics.

## Conflicts of Interest

The authors declare no conflicts of interest.

## Data Availability

Data sharing not applicable to this article as no datasets were generated or analysed during the current study.
